# Association between high-risk HPV types, HLA DRB1* and DQB1* alleles and cervical cancer in British women

**DOI:** 10.1054/bjoc.1999.1103

**Published:** 2000-03-06

**Authors:** J Cuzick, G Terry, L Ho, J Monaghan, A Lopes, P Clarkson, I Duncan

**Affiliations:** Department of Mathematics, Statistics & Epidemiology, Imperial Cancer Research Fund, 61 Lincoln's Inns Field, London, WC2A 3PX, UK; Department of Molecular Pathology, University College London, UK; Gynaecological Oncology Centre, Gateshead Hospitals NHS Trust, UK; Mayday Hospital, Thornton Heath, UK; Ninewells Hospital, UK

**Keywords:** HPV, HLA, cervical cancer

## Abstract

Cervical scrapes from 116 British women referred with cervical cancer were tested for the presence of high oncogenic risk human papillomavirus (HPV) genotypes (HPV_hr_). Ninety-four per cent of the scrapes had one or more of these virus types and 66% were HPV16-positive. HPV18 was more frequent in adenocarcinoma. No evidence was found for an increased cancer risk associated with the HPV16 E6 350G variant. The HLA DRB1* and DQB1* alleles in these women and in 155 women with normal cytology and negative for HPV_hr_DNA were compared. DQB1*0301 alone (*2P* = 0.02) and in combination with DRB1*0401 (*2P* = 0.02) was found to be associated with cervical cancer. This was more marked in cancers positive for HPV types other than HPV16. In contrast, DRB1*1501 alone and in combination with DQB1*0602 was not significantly elevated in cancers overall, but did show some excess in HPV16-positive cancers (*2P* = 0.05), associated with HPV16-positive cervical cancers. Taking all cancers together, a marginally significant protective effect was found for DQB1*0501 (*2P* = 0.03) but no protective effect could be seen for DRB1*1301. © 2000 Cancer Research Campaign


British Journal of Cancer (2000) 82(7), 1348–1352
© 2000 Cancer Research Campaign
DOI: 10.1054/ bjoc.1999.1103, available online at http://www.idealibrary.com on IDEAL®

### Association between high-risk HPV types, HLA DRB1* and DQB1* alleles and cervical cancer in British women

**J Cuzick[1], G Terry[1,2], L Ho[1,2], J Monaghan[3], A Lopes[3], P Clarkson[4] and I Duncan[5]**

[1]Department of Mathematics, Statistics & Epidemiology, Imperial Cancer Research Fund, 61 Lincoln's Inns Field, London WC2A 3PX, UK; [2]Department of Molecular Pathology, University College London, UK; [3]Gynaecological Oncology Centre, Gateshead Hospitals NHS Trust, UK; [4]Mayday Hospital, Thornton Heath, UK; [5]Ninewells Hospital, Dundee, UK

**Summary** Cervical scrapes from 116 British women referred with cervical cancer were tested for the presence of high oncogenic risk human papillomavirus (HPV) genotypes ($HPV_{hr}$). Ninety-four per cent of the scrapes had one or more of these virus types and 66% were HPV16-positive. HPV18 was more frequent in adenocarcinoma. No evidence was found for an increased cancer risk associated with the HPV16 E6 350G variant. The HLA DRB1* and DQB1* alleles in these women and in 155 women with normal cytology and negative for $HPV_{hr}$ DNA were compared. DQB1*0301 alone (*2P* = 0.02) and in combination with DRB1*0401 (*2P* = 0.02) was found to be associated with cervical cancer. This was more marked in cancers positive for HPV types other than HPV16. In contrast, DRB1*1501 alone and in combination with DQB1*0602 was not significantly elevated in cancers overall, but did show some excess in HPV16-positive cancers (*2P* = 0.05), associated with HPV16-positive cervical cancers. Taking all cancers together, a marginally significant protective effect was found for DQB1*0501 (*2P* = 0.03) but no protective effect could be seen for DRB1*1301. © 2000 Cancer Research Campaign

**Keywords:** HPV; HLA; cervical cancer

At least 15 types of high oncogenic risk human papillomavirus ($HPV_{hr}$) DNA have been detected in the uterine cervix (Lorincz et al, 1992). Most infections with these viruses regress spontaneously, but in a small proportion of women infection can become persistent and lead to the development of precancerous lesions (cervical intraepithelial neoplasia grades 2 and 3, CIN2/3 or HSIL) and ultimately cancer. Since the risk of cervical cancer is population- (IARC, 1995) and HPV type-dependent (Lorincz et al, 1992), specific viral and/or local host co-factors are believed to be necessary for the development of precancer and cancer.

Previously we found that HPV16, 18, 31 and 33 were common in CIN2/3 lesions in British women (Cuzick et al, 1994) but the prevalence of different $HPV_{hr}$ in invasive cervical cancer in the UK is not known and little Western European data contributed to the worldwide study of Bosch et al (1995). This information is required if the role of $HPV_{hr}$ in the oncogenic process is to be critically assessed in a UK context. Individual variants of a single $HPV_{hr}$ genotype may also influence the development of disease. For example, in HPV16 isolates a polymorphism (T/G) at nucleotide 350 in the E6 gene has been reported to occur more frequently as G in isolates from CIN2/3 and cervical cancer than in isolates from low-grade lesions and this is possibly population-dependent (Zehbe et al, 1998*a*). Moreover, nt350 G variants are more likely to persist at high copy number in low-grade lesions than the prototype virus (nt350T) (Londesborough et al, 1996; Xi et al, 1997). These results suggest a complex relationship between this polymorphism and the development of cervical disease; a failure in E6 antigen presentation has been suggested. The products of the HLA (human leucocyte antigen) class I and class II genes are highly polymorphic and play a major role in regulating T-cell responses to foreign antigens, including viral antigens. The particular alleles inherited by an individual may help to determine the outcome of certain viral infections. Unfortunately the immunogenetic status associated with HPV infections and HPV16 E6 variants determined so far has provided inconclusive results (Londesborough et al, 1996; Bontkes et al, 1998; Terry et al, 1998). In this study, we have assessed the prevalence of HPV16 and other $HPV_{hr}$ types in British women presenting with cervical cancer and estimated the frequencies of HPV16 E6 gene polymorphisms in isolates. The prevalence of HLA DRB1* and DQB1* alleles was estimated, compared with a control population and examined for interaction with HPV types and variants. The implications of viral and host factors in relation to the cervical cancer risk are discussed.

*Received 26 July 1999*
*Revised 22 October 1999*
*Accepted 25 October 1999*

*Correspondence to:* J Cuzick

### PATIENTS AND METHODS

### Patients

Cervical scrapes were collected from women (average age ± 1 s.d. = 50.18 ± 15.81 years) on referral with cervical cancer prior to treatment from clinics in Britain. The first 116 scrapes found positive for β-globin DNA by polymerase chain reaction (PCR) were tested blind. Only histological data were made available to the study. The scrapes were collected from Scotland (1), North England (87), Midlands (1) and South-east England (27). One hundred and fifty-five scrapes collected from normal women as part of a previous study (Cuzick et al, 1999) from which both DRB1* and DQB1* alleles could be determined were used as matched controls in the HLA analysis. These women had normal cytology and were negative for $HPV_{hr}$ as tested by consensus PCR/SHARP assay (Digene Corp.) and by HPV16, 18, 31, 33, 35, 58 type-specific PCR (Cuzick et al, 1999).

**Table 1** Prevalence of HR-HPV genotypes in British women diagnosed with cervical cancer

| Histology | No. tested | No. with HR-HPV | | | | | | No. without HR-HPV |
|---|---|---|---|---|---|---|---|---|
| | | Any HR-HPV | HPV16 | HPV18 | HPV31 | HPV33 | Others | |
| Adenocarcinoma | 19 | 16 | 8 | 6 | 2 | 0 | 0 | 3 |
| Adeno-squamous carcinoma | 12 | 10 | 6 | 2 | 2 | 0 | 0 | 2 |
| Squamous carcinoma | 85 | 83 | 62 | 4 | 7 | 5 | 5 | 2 |
| Total (%) | 116 (100) | 109 (94) | 76 (66) | 12 (10) | 11 (9) | 5 (4) | 5 (4) | 7 (6) |

Six double infections with HPV16 and one other HR-HPV type are counted only once under HPV16.

### DNA preparation

Cells from cervical scrapes were collected in sterile phosphate-buffered saline (PBS) containing antibiotics and fungizone. After centrifugation, each pellet was re-suspended and heated to 98°C in 1 ml of sterile PBS for 30 min. Two microliters of the supernatant was used for either HPV or HLA PCR.

### Consensus and type specific PCR for HPV16, 18, 31 and 33

This was carried out as previously described (Cuzick et al, 1999).

### HPV16 E6 sequencing

HPV16 E6 sequence was amplified (by nested PCR) as previously described by Wheeler et al (1997). The quality of the PCR fragments was assessed visually under UV after electrophoresis in agarose gels. The templates were purified using Wizard PCR Preps (Promega Corp.). Sequencing reactions were carried out using chain termination sequencing reactions and Thermo-sequenase (Amersham Pharmacia Biotech). The following Cy5-labelled sequencing primers were used:

Forward: 5′-ATAAAAGCAGACAT-3′
Reverse: 5′-TGTAGGTGTATCTCC-3′.

Reactions were analysed using an automated sequencer (AlfExpress, Amersham Pharmacia Biotech). HPV16 E6 variants were identified and confirmed by direct sequencing in both directions between nucleotides 80 and 567. HPV16r nucleic acid sequence was used as reference. Sixty-four of the 76 HPV16 isolates yielded identical sequencing results from both directions and only these were included in the study.

### HLA typing

This was carried out by direct sequencing of amplified DRB1* and DQB1* alleles as previously described (Terry et al, 1998). Haplotypes were inferred based on known patterns of linkage disequilibrium in Whites for these loci (Apple et al, 1994; Odunsi et al, 1996).

### Statistical analysis

All *P*-values used for comparing the number of cancers and controls who were positive for a specific allele or haplotype were two-sided Fisher exact values (obtained from doubling one-sided values). All confidence intervals (CI) are 95% coverage intervals. The analysis was carried out using the SAS statistics package.

### RESULTS

### HPV genotypes

The HPV genotypes associated with cancer of different histology are shown in Table 1. Of the 116 cancer samples tested, 109 (94%) were found to be positive for HR types. Six lesions contained two HPV types. HPV16 was found in 66% of all cancers and was far more common than other types (HPV18 10%, HPV31 9%, HPV33 4% and other $HPV_{hr}$ types 4%) (Table 1).

### HPV16 polymorphism in E6

The sequencing results are shown in Table 2. HPV subtype designations are as given by Yamada et al (1995). Nt350T subtypes were found in 58% of all HPV16-positive cancers (31 had E-350T, three had AF) while 42% of isolates had nt350 G (16 had E-350 G, two had E131 G, five had AA and two had NA1). This is not significantly different from our previous study where nt350T and nt350G subtypes were found respectively in 57% and 43% of 14 women who had a normal cervix at both initial and follow-up visits (Londesborough et al, 1996).

### HLA alleles and haplotypes

Table 3 summarizes the data for the HLA DQB1* and DRB1* alleles from the 116 cancers and 155 controls which occur at frequencies equal to or greater than 2% in the control population. HLA typing by nucleic acid sequencing as reported here is not carried out routinely in the UK and it is not known if the allelic frequencies observed in our control population are representative of the general British population. Frequencies of common alleles (those which occur at ≥ 10%) are similar to those previously reported for the UK female population (Duggan-Keen et al, 1996; Odunsi et al, 1996). DQB1*0301 ($2P$ = 0.02, odds ratio (OR) = 1.67, CI = 1.06–2.65) was found to be associated with cervical cancer and this association is stronger in cancers positive for HPV types other than HPV16 ($2P$ = 0.006, OR = 2.54, CI = 1.30–4.92). In contrast, DQB1*1501 was not elevated in cancer overall, but a relationship with HPV16-positive cancer was suggestive ($2P$ = 0.05, OR = 1.73, CI = 0.99–3.03). A marginally significant protective effect was found for DQB1*0501 ($2P$ = 0.03, OR = 0.53, CI = 0.29–0.96), but not for DRB1*1301. No significant change in cancer risk was detected in women homozygous for any of these alleles.

An analysis of the DRB1*/DQB1* assigned haplotypes occurring at frequencies equal to or greater than 2% in the controls (Table 3) identified six frequently occurring DRB1*/DQB1*

**Table 2** HPV16 E6 polymorphic sites in isolates from cervical cancers

| HPV16 subtype | No. of templates | % of total | E6 nucleotides | | | | | | | | | | | | | |
|---|---|---|---|---|---|---|---|---|---|---|---|---|---|---|---|---|
| | | | 131 | 132 | 143 | 145 | 183 | 257 | 286 | 289 | 310 | 314 | 335 | 350 | 369 | 532 |
| Prototype | | | A | G | C | G | T | A | T | A | T | T | C | T | A | A |
| E-350T | 28 | 53 | | | | | | | | | | | | | | |
| | 1 | | | | | | | | G | | | | | | | |
| | 1 | | | | | | | | | | | | | | G | |
| | 1 | | | | | | | | | | G | | | | | |
| E-350G | 14 | 27 | | | | | | | | | | | | G | | |
| | 1 | | | | | | | G | | | | | | G | | |
| | 1 | | | | | | | | | | | G | | G | | |
| E131G | 2 | 3 | G | | | | | | | | | | | G | | |
| AF | 1 | 5 | | C | G | T | | | G | G | | | T | | | |
| | 2 | | | C | G | T | | | A | G | | | T | | | |
| AA | 3 | 8 | | | | T | | | A | G | | | T | G | | G |
| | 1 | | | | | | | | A | G | | | T | G | | G |
| | 1 | | | | | T | G | | A | G | | | T | G | | G |
| NA1 | 2 | 3 | | | | T | | | A | G | | | T | G | | |

HPV subtype designations are from Wheeler et al (1997).

haplotypes in our control population – 0101/0501, 0301/0201, 0401/0301, 0701/0201, 1101/0301 and 1501/0602. Only 0401/0301 showed a clear association with cervical cancers taken as a whole ($2P$ = 0.02, OR = 2.40, CI = 1.12–5.22). This same haplotype was also found to be associated with cancers positive for $HPV_{hr}$ types other than HPV16 ($2P$ = 0.001, OR = 4.75, CI = 1.84–12.21) in keeping with the results for the constituent alleles. In contrast, DRB1*/DQB1* haplotype 1501/0602 was found to be weakly associated only with cancers positive for HPV16 ($2P$ = 0.05, OR = 1.81, CI = 1.00–3.29).

### The association of HPV16 European isolates polymorphic at E6 nt350 with DRB1* and DQB1* alleles

No significant differences in the HLA frequencies in HPV16 E350G/T variants could be found, possibly due to limitations in sample size. However the T variant occurred more frequently than the G variant in women with DRB1*1501 (15 T to 4 G, $2P$ = 0.17), DQB1*0602 (17 T to 5 G, $2P$ = 0.15) alleles, or with the corresponding haplotype 1501/0602 (15 T to 3 G, $2P$ = 0.07) which suggests this might be worth studying further.

### DISCUSSION

$HPV_{hr}$ was detected in 94% of the scrapes from women who had cervical cancer and 66% were HPV16 (Table 1). The percentage of $HPV_{hr}$ genotypes which are HPV16 observed in cancer is markedly different from that in the normal British screening population aged over 35 years (Cuzick et al, 1999). This indicates that HPV16 is not only more prevalent than other $HPV_{hr}$ types in the UK, but is also more oncogenic. How the oncogenic process relates to an evasion of the host immune surveillance mechanism is not clear.

The immunogenetics of HLA DRB1* and DQB1* in association with HPV16 and other $HPV_{hr}$ positive cervical intra-epithelial neoplasms (CIN) and cancer has been investigated in many studies but the results obtained so far have been inconsistent. This has usually been attributable to population differences or to whether cytology, serology or nucleic acid-based methods were used to determine the HPV and/or HLA status (Wank et al, 1992; Apple et al, 1994, 1995; Duggan-Keen et al, 1996; Odunsi et al, 1996; Sanjeevi et al, 1996; Bontkes et al, 1998; Helland et al, 1998; Hildesheim et al, 1998), but chance association among the multiple comparisons performed may also be an important factor. We have used well-characterized, consistent analytical methods throughout this study. $HPV_{hr}$ genotypes were determined by both type-specific PCR and consensus PCR/SHARP using probes for $HPV_{hr}$ types. This is to ensure vigorously that all women in the control population are $HPV_{hr}$-negative. HPV16 E6 sequences and HLA types were determined precisely by nucleic acid sequencing. However, chance is still an important source of error especially for comparisons between $HPV_{hr}$ types where the numbers are small. Also there is a geographic disparity between our cancer cases (mostly northern England) and controls (mostly southern England). Since the frequencies of the commonly occurring alleles in our normal control population reported here are consistent with those we reported previously for women from southern England (Odunsi et al, 1996) and to those reported for northern England (Duggan-Keen et al, 1996), the gene pools appear to be similar.

The most commonly reported HLA alleles in association with cervical cancer overall is DQB1* 03 (Wang et al, 1992) and we also obtained the most significant findings for this allele. In this study, we identified the most important at-risk allele as DQB1*0301 (OR = 1.67, CI = 1.06–2.65) with the risk of cancer being further increased when DRB1*0401 is also present. We have previously found these same alleles to be associated respectively with HPV16-, 18-, 31- and 33-positive CIN lesions from the UK (DQBI*0301 OR = 2.77, DRB1*0401 OR = 2.34, 0401/0301 OR = 2.88) (Odunsi et al, 1996). In a Puerto Rican population, Hildesheim et al, (1998) associated HPV16-positive lesions with DQB1*0302. It seems clear that DQB1*03 plays an important role in the pathogenesis of cervical cancer and CIN2/3. In contrast, DQB1*0501 but not DRB1*1301 was found to have the protective effect (Table 3) found by other workers (Apple et al, 1994; Odunsi et al, 1996). Failure to find a significant association with DRB1*1301 may be due to the rarity of the allele in our sample and to the relatively small sample size.

*© 2000 Cancer Research Campaign*

**Table 3** Significant HLA DQB1 and DRB1 alleles and assigned haplotypes in all cervical cancers and in those with either HPV16 or other HR-HPV types.

| HLA | Alleles or Haplotypes | Normal | | All cancers | | | | | HPV16-positive cancers | | | | | Other HR-HPV-positive cancers | | | | |
|---|---|---|---|---|---|---|---|---|---|---|---|---|---|---|---|---|---|---|
| | | n | f | n | f | 2*P* | OR | CI | n | f | 2*P* | OR | CI | n | f | 2*P* | OR | CI |
| DQB1* | 0201 | 76 | 0.245 | 59 | 0.261 | 0.89 | | | 36 | 0.240 | 1.0 | | | 16 | 0.258 | 0.94 | | |
| | 0301 | 49 | 0.158 | 54 | 0.239 | 0.02 | 1.67 | 1.06–2.65 | 33 | 0.220 | 0.14 | | | 20 | 0.323 | 0.006 | 2.54 | 1.30–4.92 |
| | 0302 | 20 | 0.065 | 16 | 0.071 | 0.87 | | | 8 | 0.053 | 0.81 | | | 8 | 0.129 | 0.14 | | |
| | 0303 | 8 | 0.026 | 13 | 0.058 | 0.09 | | | 9 | 0.060 | 0.13 | | | 4 | 0.065 | 0.24 | | |
| | 0501 | 46 | 0.148 | 19 | 0.084 | 0.03 | 0.53 | 0.29–0.96 | 13 | 0.087 | 0.08 | | | 4 | 0.065 | 0.10 | | |
| | 0503 | 14 | 0.045 | 5 | 0.022 | 0.05 | | | 5 | 0.033 | 0.75 | | | 0 | 0.000 | 0.14 | | |
| | 0601 | 18 | 0.058 | 7 | 0.031 | 0.14 | | | 6 | 0.040 | 0.56 | | | 0 | 0.000 | 0.07 | | |
| | 0602 | 49 | 0.158 | 40 | 0.177 | 0.69 | | | 32 | 0.213 | 0.18 | | | 6 | 0.097 | 0.29 | | |
| | 0603 | 13 | 0.042 | 6 | 0.027 | 0.32 | | | 4 | 0.027 | 0.60 | | | 1 | 0.016 | 0.58 | | |
| | All alleles | 310 | 1.000 | 226 | 1.000 | | | | 150 | 1.000 | | | | 62 | 1.000 | | | |
| DRB1* | 0101 | 27 | 0.087 | 16 | 0.074 | 0.75 | | | 13 | 0.092 | 0.88 | | | 1 | 0.016 | 0.09 | | |
| | 0102 | 7 | 0.023 | 3 | 0.014 | 0.44 | | | 3 | 0.021 | 1.00 | | | 0 | 0.000 | 0.62 | | |
| | 0301 | 41 | 0.132 | 35 | 0.161 | 0.73 | | | 17 | 0.120 | 0.80 | | | 11 | 0.180 | 0.43 | | |
| | 0401 | 27 | 0.087 | 29 | 0.134 | 0.06 | | | 14 | 0.099 | 0.86 | | | 15 | 0.246 | 0.002 | 3.42 | 1.59–7.29 |
| | 0701 | 37 | 0.119 | 30 | 0.138 | 0.75 | | | 23 | 0.162 | 0.34 | | | 7 | 0.115 | 1.0 | | |
| | 0801 | 9 | 0.029 | 1 | 0.005 | 0.36 | | | 1 | 0.007 | 0.30 | | | 0 | 0.000 | 0.44 | | |
| | 1101 | 19 | 0.061 | 11 | 0.051 | 0.61 | | | 7 | 0.049 | 0.75 | | | 3 | 0.049 | 0.97 | | |
| | 1301 | 13 | 0.042 | 5 | 0.023 | 1.0 | | | 4 | 0.028 | 0.80 | | | 0 | 0.000 | 0.22 | | |
| | 1302 | 8 | 0.026 | 4 | 0.018 | 0.73 | | | 4 | 0.028 | 0.99 | | | 0 | 0.000 | 0.52 | | |
| | 1401 | 10 | 0.032 | 4 | 0.018 | 0.17 | | | 4 | 0.028 | 1.0 | | | 0 | 0.000 | 0.31 | | |
| | 1501 | 40 | 0.129 | 38 | 0.175 | 0.17 | | | 29 | 0.204 | 0.048 | 1.73 | 0.99–3.03 | 6 | 0.098 | 0.69 | | |
| | All alleles | 310 | 1.000 | 217 | 1.000 | | | | 142 | 1.000 | | | | 61 | 1.000 | | | |
| DRB1*/DQBI* | 0101/0501 | 25 | 0.087 | 11 | 0.053 | 0.21 | | | 9 | 0.063 | 0.52 | | | 1 | 0.017 | 0.08 | | |
| | 0301/0201 | 38 | 0.132 | 32 | 0.155 | 0.54 | | | 17 | 0.120 | 0.85 | | | 10 | 0.167 | 0.60 | | |
| | 0401/0301 | 13 | 0.045 | 21 | 0.102 | 0.02 | 2.40 | 1.12–5.22 | 10 | 0.070 | 0.38 | | | 11 | 0.183 | 0.001 | 4.75 | 1.84–12.21 |
| | 0701/0201 | 27 | 0.094 | 15 | 0.073 | 0.51 | | | 13 | 0.092 | 1.0 | | | 2 | 0.033 | 0.18 | | |
| | 1101/0301 | 11 | 0.038 | 12 | 0.058 | 0.41 | | | 7 | 0.049 | 0.76 | | | 4 | 0.067 | 0.50 | | |
| | 1501/0602 | 33 | 0.115 | 33 | 0.160 | 0.18 | | | 27 | 0.190 | 0.05 | 1.81 | 1.00–3.29 | 5 | 0.083 | 0.65 | | |
| | All haplotypes | 288 | 1.000 | 206 | 1.000 | | | | 142 | 1.000 | | | | 60 | 1.000 | | | |

Only alleles and haplotypes with frequencies ≥ 2% in the control population are shown. Associations with cervical cancer by HPV group. [box] = Significant positive associations at $2P = 0.05$ (Fisher exact, two-sided)

© 2000 Cancer Research Campaign

Consistent with a previous report (Apple et al, 1994), our results show a small risk of DRB1*1501 allele (OR = 1.73) and the DRB1*/DQB1* haplotype 1501/0602 (OR = 1.81) in women with HPV16-positive cancers (Table 3). This estimate is smaller than the high cancer risk (OR = 4.78) found for this haplotype in a study of Hispanic women (Apple et al, 1994). In addition we found this haplotype occurring fivefold more frequently in women with cervical cancers positive for the E350T rather than E350G variant, although the statistical significance has yet to be confirmed ($2P = 0.058$).

As in the case of cervical cancers overall, we found cancers positive for HPV types other than HPV16 to be strongly associated with DRB1*0401 and DQB1*0301 alleles and the corresponding haplotype. CIN lesions positive for these HPV types have also been associated with DQB1*0301 (Odunsi et al, 1994; Sanjeevi et al, 1996).

In conclusion, we find that in a British population virtually all cervix cancers are HPV-positive and HPV16 is by far the most common type. The HLA DQB1*0301 allele confers an increased risk for cervical cancer regardless of the type of HPV involved. There is some evidence that HLA DRB1*/DQB1* haplotype 1501/0602 confers an increased risk, possibly restricted to HPV16-positive cervical cancer. A very large population-matched study of women with and without cancer is needed to fully evaluate any risk related to interactions between HPV type or sequence variants and HLA alleles.

### REFERENCES

Apple RJ, Erlich HA, Klitz W, Manos MM, Becker TM and Wheeler CM (1994) HLA DR-DQ associations with cervical carcinoma show papillomavirus-type specificity. *Nat Genet* **6**: 157–162

Bontkes HJ, Van Duin M, De Gruij TD, Duggan-Keen MF, Walboomers JMM, Stukart MJ, Verheijen RHM, Helmerhorst TJM, Meijer CJLM, Scheper RJ, Stevens FRA, Dyer PA, Sinnott P and Stern PL (1998) HPV16 infection and progression of cervical intra-epithelial neoplasia: analysis of HLA polymorphism and HPV16 E6 sequence variants. *Int J Cancer* **78**: 166–171

Bosch FX, Manos MM, Munoz N, Sherman M, Jansen AM, Peto J, Schiffman MH, Moreno V, Kurman R and Shah KV (1995) Prevalence of human papillomavirus in cervical cancer: a worldwide perspective. *J Natl Cancer Inst* **87**: 78–84

Cuzick J, Beverley E, Ho L, Terry G, Sapper K, Mielzynska I, Lorincz A, Chan W-K, Krausz P and Soutter P (1999) HPV testing in primary screening of older women. *Br J Cancer* **81**: 554–558

Duggan-Keen MF, Keating PJ, Stevens FRA, Sinnott P, Snijders PLF, Walboomers JMM, Davidson S, Hunter RD, Dyer PA and Stern PL (1996) Immunogenetic factors in HPV-associated cervical cancer: influence on disease progression. Eur J Immunogen **23**: 275–284

Hildesheim A, Schiffman MH, Gravitt PE, Glass AG, Greer CE, Zhang T, Scott DR, Rush BB, Lawler P, Sherman ME, Kurman RJ and Manos MM (1994) Persistence of type-specific human papillomavirus infection among cytologically normal women. *J Infect Dis* **169**: 235–240

Hildesheim A, Schiffman M, Scott DR, Marti D, Kissner T, Sherman ME, Glass AG, Manos MM, Lorincz AT, Kurman RJ, Buckland J, Rush BB and Carrington M (1998) Human leukocyte antigen class I/II alleles and development of human papillomavirus-related cervical neoplasia: Results from a case-control study conducted in the United States. *Can Epidemiol Biomarkers Prev* **7**: 1035–1041

IARC (1995) Human papillomavirus IARC monographs on the evaluation of carcinogenic risks to humans, Vol. 64. IARC: Lyon

Londesborough P, Ho L, Terry G, Cuzick J, Wheeler C and Singer A (1996) Human papillomavirus genotype as a predictor of persistence and development of high-grade lesions in women with minor cervical abnormalities. *Int J Cancer* **69**: 364–368

Lorincz AT, Reid R, Jenson AB, Greenberg MD, Lancaster W and Kurman RJ (1992) Human papillomavirus infection of the cervix: relative risk associations of 15 common anogenital types. *Obs Gynaecol* **79**: 328–337

Nindl I, Rindfleisch K, Lotz B, Schneider A and Durst (1999) Uniform distribution of HPV16 E6 and E7 variants in patients with normal histology, cervical intra-epithelial neoplasia and cervical cancer. *Int J Cancer* **82**: 203–207

Odunsi K, Terry G, Ho L, Bell J, Cuzick J and Ganesan TS (1996) Susceptibility to human papillomavirus-associated cervical intra-epithelial neoplasia is determined by specific HLA DR-DQ alleles. *Int J Cancer* **67**: 595–602

Sanjeevi CB, Hjelmstrom P, Hallmans G, Wiklund F, Lenner P, Angstrom T, Dillner J and Lernmark A (1996) Different HLA-DR-DQ haplotypes are associated with cervical intraepithelial neoplasia among human papillomavirus type-16 seropositive and seronegative Swedish women. *Int J Cancer* **68**: 409–414

Terry G, Ho L and Cuzick J (1998) Analysis of E2 amino acid variants of human papillomavirus types 16 and 18 and their associations with lesion grade and HLA DR/DQ type. *Int J Cancer* **73**: 651–655

Wank R, Schendel DJ and Thomssen C (1992) HLA antigens and cervical cancer. *Nature* **356**: 22–23

Wheeler CM, Yamada T, Hildesheim A and Jenison SA (1997) Human papillomavirus type 16 sequence variants; identification by E6 and L1 lineage-specific hybridization. *J Clin Microbiol* **35**: 11–19

Xi LF, Koustsky LA, Galloway DA, Kuypers J, Hughes JP, Wheeler CM, Holmes KK and Kiviat NB (1997) Genomic variation of human papillomavirus type 16 and risk for high grade cervical intraepithelial neoplasia. *J Natl Cancer Inst* **89**: 796–802

Yamada T, Wheeler CM, Halpern AL, Stewart ACM, Hildesheim A and Jenison SA (1995) Human papillomavirus type 16 variant lineages in United States populations characterised by nucleotide sequence analysis of the E6, L2 and L1 coding segments. *J Virol* **69**: 7743–7753

Zehbe I, Voglino G, Delius H, Wilander E and Tommasino M (1998*a*). Risk of cervical cancer and geographical variations of human papillomavirus 16 E6 polymorphisms. *Lancet* **352**: 1441–1442

Zehbe I, Wilander E, Delius H, Tommasino M (1998*b*). Human papillomavirus 16 E6 variants are more prevalent in invasive cervical carcinoma than the prototype. *Cancer Res* **58**: 829–833

 *© 2000 Cancer Research Campaign*
